# Supplementary material for: Unveiling the Bioactive Potential of the Invasive Jellyfish Phyllorhiza punctata Through Integrative Transcriptomic and Proteomic Analyses
Source: Biomolecules. 2025 Aug 4;15(8):1121. doi: 10.3390/biom15081121 (PMC12383608; doi:10.3390/biom15081121)
Supplement: Supplementary file 1 [file biomolecules-15-01121-s001.zip › Suplementary Material/FileS3/PEPFOLD4/XP_065054292_5353/clusters.html]

 PEP-FOLD clusters


Download archive of all models  here   

|  |  |  |
| --- | --- | --- |
| # | file | sOPEP |
| # cluster 1 - Size: 43 |  |  |
|  | XP\_065054292\_5353-model1 | -7.18534 |
|  | XP\_065054292\_5353-model1.1 | -6.6974 |
|  | XP\_065054292\_5353-model1.2 | -6.6974 |
|  | XP\_065054292\_5353-model1.3 | -6.6974 |
|  | XP\_065054292\_5353-model1.4 | -6.5547 |
|  | XP\_065054292\_5353-model1.5 | -6.47329 |
|  | XP\_065054292\_5353-model1.6 | -6.29388 |
|  | XP\_065054292\_5353-model1.7 | -6.29388 |
|  | XP\_065054292\_5353-model1.8 | -6.22823 |
|  | XP\_065054292\_5353-model1.9 | -6.22823 |
|  | XP\_065054292\_5353-model1.10 | -6.22823 |
|  | XP\_065054292\_5353-model1.11 | -6.22823 |
|  | XP\_065054292\_5353-model1.12 | -6.19501 |
|  | XP\_065054292\_5353-model1.13 | -6.18153 |
|  | XP\_065054292\_5353-model1.14 | -6.0951 |
|  | XP\_065054292\_5353-model1.15 | -6.0951 |
|  | XP\_065054292\_5353-model1.16 | -6.0748 |
|  | XP\_065054292\_5353-model1.17 | -6.05498 |
|  | XP\_065054292\_5353-model1.18 | -6.05498 |
|  | XP\_065054292\_5353-model1.19 | -6.05498 |
|  | XP\_065054292\_5353-model1.20 | -6.05498 |
|  | XP\_065054292\_5353-model1.21 | -6.05498 |
|  | XP\_065054292\_5353-model1.22 | -6.05498 |
|  | XP\_065054292\_5353-model1.23 | -6.05498 |
|  | XP\_065054292\_5353-model1.24 | -6.03599 |
|  | XP\_065054292\_5353-model1.25 | -5.97057 |
|  | XP\_065054292\_5353-model1.26 | -5.92066 |
|  | XP\_065054292\_5353-model1.27 | -5.92066 |
|  | XP\_065054292\_5353-model1.28 | -5.92066 |
|  | XP\_065054292\_5353-model1.29 | -5.92066 |
|  | XP\_065054292\_5353-model1.30 | -5.92066 |
|  | XP\_065054292\_5353-model1.31 | -5.92066 |
|  | XP\_065054292\_5353-model1.32 | -5.92066 |
|  | XP\_065054292\_5353-model1.33 | -5.92066 |
|  | XP\_065054292\_5353-model1.34 | -5.91784 |
|  | XP\_065054292\_5353-model1.35 | -5.88516 |
|  | XP\_065054292\_5353-model1.36 | -5.8287 |
|  | XP\_065054292\_5353-model1.37 | -5.70537 |
|  | XP\_065054292\_5353-model1.38 | -5.18502 |
|  | XP\_065054292\_5353-model1.39 | -3.5364 |
|  | XP\_065054292\_5353-model1.40 | -3.50445 |
|  | XP\_065054292\_5353-model1.41 | -3.49343 |
|  | XP\_065054292\_5353-model1.42 | -3.33642 |
| # cluster 2 - Size: 1 |  |  |
|  | XP\_065054292\_5353-model2 | -6.27128 |
| # cluster 3 - Size: 1 |  |  |
|  | XP\_065054292\_5353-model3 | -4.94198 |
| # cluster 4 - Size: 3 |  |  |
|  | XP\_065054292\_5353-model4 | -4.30984 |
|  | XP\_065054292\_5353-model4.1 | -3.55637 |
|  | XP\_065054292\_5353-model4.2 | -3.40058 |
| # cluster 5 - Size: 1 |  |  |
|  | XP\_065054292\_5353-model5 | -3.5877 |
| # cluster 6 - Size: 4 |  |  |
|  | XP\_065054292\_5353-model6 | -3.51522 |
|  | XP\_065054292\_5353-model6.1 | -3.38432 |
|  | XP\_065054292\_5353-model6.2 | -3.23778 |
|  | XP\_065054292\_5353-model6.3 | -2.84214 |
| # cluster 7 - Size: 6 |  |  |
|  | XP\_065054292\_5353-model7 | -3.45691 |
|  | XP\_065054292\_5353-model7.1 | -3.38584 |
|  | XP\_065054292\_5353-model7.2 | -3.31724 |
|  | XP\_065054292\_5353-model7.3 | -3.30634 |
|  | XP\_065054292\_5353-model7.4 | -3.30634 |
|  | XP\_065054292\_5353-model7.5 | -3.06804 |
| # cluster 8 - Size: 2 |  |  |
|  | XP\_065054292\_5353-model8 | -3.22703 |
|  | XP\_065054292\_5353-model8.1 | -3.01333 |
| # cluster 9 - Size: 4 |  |  |
|  | XP\_065054292\_5353-model9 | -3.02565 |
|  | XP\_065054292\_5353-model9.1 | -3.02252 |
|  | XP\_065054292\_5353-model9.2 | -2.90402 |
|  | XP\_065054292\_5353-model9.3 | -2.87727 |
| # cluster 10 - Size: 7 |  |  |
|  | XP\_065054292\_5353-model10 | -3.01758 |
|  | XP\_065054292\_5353-model10.1 | -2.94754 |
|  | XP\_065054292\_5353-model10.2 | -2.94054 |
|  | XP\_065054292\_5353-model10.3 | -2.94047 |
|  | XP\_065054292\_5353-model10.4 | -2.92114 |
|  | XP\_065054292\_5353-model10.5 | -2.85348 |
|  | XP\_065054292\_5353-model10.6 | -2.84054 |
| # cluster 11 - Size: 5 |  |  |
|  | XP\_065054292\_5353-model11 | -2.98623 |
|  | XP\_065054292\_5353-model11.1 | -2.98082 |
|  | XP\_065054292\_5353-model11.2 | -2.95746 |
|  | XP\_065054292\_5353-model11.3 | -2.89192 |
|  | XP\_065054292\_5353-model11.4 | -2.8083 |
| # cluster 12 - Size: 8 |  |  |
|  | XP\_065054292\_5353-model12 | -2.93415 |
|  | XP\_065054292\_5353-model12.1 | -2.92488 |
|  | XP\_065054292\_5353-model12.2 | -2.90847 |
|  | XP\_065054292\_5353-model12.3 | -2.89394 |
|  | XP\_065054292\_5353-model12.4 | -2.85589 |
|  | XP\_065054292\_5353-model12.5 | -2.82982 |
|  | XP\_065054292\_5353-model12.6 | -2.82723 |
|  | XP\_065054292\_5353-model12.7 | -2.81669 |
| # cluster 13 - Size: 1 |  |  |
|  | XP\_065054292\_5353-model13 | -2.93229 |
| # cluster 14 - Size: 1 |  |  |
|  | XP\_065054292\_5353-model14 | -2.91184 |
| # cluster 15 - Size: 2 |  |  |
|  | XP\_065054292\_5353-model15 | -2.88972 |
|  | XP\_065054292\_5353-model15.1 | -2.84181 |
| # cluster 16 - Size: 2 |  |  |
|  | XP\_065054292\_5353-model16 | -2.86118 |
|  | XP\_065054292\_5353-model16.1 | -2.84264 |
| # cluster 17 - Size: 3 |  |  |
|  | XP\_065054292\_5353-model17 | -2.85975 |
|  | XP\_065054292\_5353-model17.1 | -2.84707 |
|  | XP\_065054292\_5353-model17.2 | -2.80394 |
| # cluster 18 - Size: 3 |  |  |
|  | XP\_065054292\_5353-model18 | -2.84912 |
|  | XP\_065054292\_5353-model18.1 | -2.82746 |
|  | XP\_065054292\_5353-model18.2 | -2.82746 |
| # cluster 19 - Size: 1 |  |  |
|  | XP\_065054292\_5353-model19 | -2.83092 |
| # cluster 20 - Size: 1 |  |  |
|  | XP\_065054292\_5353-model20 | -2.76208 |
| # cluster 21 - Size: 1 |  |  |
|  | XP\_065054292\_5353-model21 | -2.76091 |
|  |  |  |
